# How well do patients pursuing anti‐amyloid antibody therapies for Alzheimer's disease adhere to guidelines for promoting brain health?

**DOI:** 10.1002/alz70858_106775

**Published:** 2025-12-25

**Authors:** Kayla Riera, Seth A Gale, George Ghorayeb, Brittany M McFeeley, Kirk R Daffner

**Affiliations:** ^1^ Brigham and Women's Hospital, Boston, MA, USA

## Abstract

**Background:**

Patients seeking anti‐amyloid antibody therapies (AATs) are prepared to invest considerable time and effort to slow the progression of AD. How well these highly motivated patients follow consensus guidelines regarding modifiable risk factors for cognitive decline and dementia remains to be determined.

**Method:**

101 patients with MCI (*n* = 63) or mild dementia (*n* = 38) due to AD (mean age=74; 54% female), being evaluated for AATs were assessed on 15 modifiable risk factors by questionnaire and review of electronic medical record (EMR) data. The 15 factors included physical activity, cognitive activity, social engagement, mood, hearing, LDL cholesterol level, and HbA1c, among others. Scores (0‐2) for each factor reflected degree of adherence to recommended guidelines: 0=not met; 1=partially met/room for growth; 2=optimally met; maximum score=30 for overall adherence to brain health recommendations.

**Result:**

For 7 factors, ^3^38% of patients were not optimally following brain health guidelines: blood pressure (44%), HbA1c (59%), BMI (58%), physical activity (64%), LDL (41%), mood (53%), and cognitive activity (38%). For 4 factors, 64‐73% of patients were optimally following guidelines: sleep (64%), hearing (65%), diet (72%), and social engagement (73%). For 4 factors, >80% were optimally following guidelines: smoking (99%), alcohol (88%), vision (90%), and finding purpose in life (81%). Numerically, MCI patients exhibited slightly higher adherence scores than patients with mild dementia (*p* = 0.1). No recent data (within 1.5 years) were found in the EMR for LDL in 27% of patients and for HbA1c in 45% of patients.

**Conclusion:**

A mix of pharmacologic and lifestyle interventions will likely result in the best outcomes in AD. In this sample of very motivated AD patients pursuing AATs, a sizeable portion were not fully adhering to brain health guidelines for reducing risk of cognitive decline. Many of the factors not optimally followed are also recommended for vascular health. These results highlight a critical gap in enhancing care for patients with underlying AD. Healthcare systems must establish more effective strategies and concrete interventions for addressing modifiable risk factors. Moreover, better systems are needed to ensure relevant, actionable patient data related to brain health (e.g., HbA1c, LDL) are collected and updated in the EMR.

